# Stress Estimation Using Digital Image Correlation with Compensation of Camera Motion-Induced Error

**DOI:** 10.3390/s19245503

**Published:** 2019-12-12

**Authors:** Junhwa Lee, Seunghoo Jeong, Young-Joo Lee, Sung-Han Sim

**Affiliations:** 1 School of Urban and Environmental Engineering, Ulsan National Institute of Science and Technology (UNIST), Ulsan 44919, Korea; lee.junhwa@unist.ac.kr (J.L.); shjeong@unist.ac.kr (S.J.); 2 School of Civil, Architectural Engineering, and Landscape Architecture, Sungkyunkwan University, Suwon 16419, Korea

**Keywords:** digital image correlation, hole-drilling method, stress estimation

## Abstract

Measurement of stress levels from an in-service structure can provide important and useful information regarding the current state of a structure. The stress relaxation method (SRM) is the most conventional and practical method, which has been widely accepted for measuring residual stresses in metallic materials. The SRM showed strong potential for stress estimation of civil engineering structures, when combined with digital image correlation (DIC). However, the SRM/DIC methods studied thus far have practical issues regarding camera vibration during hole drilling. To minimize the error induced by the camera motion, the imaging system is installed at a distance from the specimen resulting in the low pixel density and the large extent of the inflicted damage. This study proposes an SRM/DIC-based stress estimation method that allows the camera to be removed during hole drilling and relocated to take the after-drilling image. Since the imaging system can be placed as close to the specimen as possible, a high pixel density can be achieved such that subtle displacement perturbation introduced by a small damage can be acquired by DIC. This study provides a detailed mathematical formulation for removing the camera relocation-induced false displacement field in the DIC result. The proposed method is validated numerically and experimentally.

## 1. Introduction

Residual stress is the remaining internal stress without applied external forces that any solid material, ranging from a microstructure to a full-scale civil infrastructure, can experience during its entire lifetime. Under presence of confining boundary condition, the residual stress can be formed when the material keeps expanding or contracting due to thermal or mechanical reasons [1]. This residual stress can be intentionally introduced to enhance material property and prolong the lifespan of the final product, therefore the industry has developed numerous strengthening mechanisms, such as laser shock peening [2], martensite [3], and prestress [4]. On the other hand, unfavorable and unintended residual stresses in a structure consistently take portion of stress level that the structure can accommodate, which ends in service life reduction or system failure for the worst case. Such a well-known problem is a buckling phenomenon in continuous welded rail subjected to thermal expansion under hot weather that leaves the track vulnerable to bend even with a slight curve. A variety of structures can experience favorable or unfavorable residual stresses; thus, appropriate measurement techniques are desired.

The residual stress relaxation method (SRM) [5,6] is one of the most traditional and effective ways of residual stress estimation. Upon inflicting a small damage (e.g., hole) on the surface of a material, the stress relaxation around the damage induces deformation, of which level and pattern has a linear relationship with the residual stresses existing in the material. The finite element model [7] or analytical solution based on the Kirsch equations [8,9] can be used to relate the deformation and the residual stresses. In general, strain gauges are employed in SRM to measure the deformation around the inflicted damage, in turn, to estimate stress level. Due to the simple and intuitive preparation of the strain rosette, a number of studies have validated the strain gauge-based method through practical applications in the laboratory environment [10,11,12,13,14,15,16] that even detailed guidelines are extensively documented in the ASTM standard [17] with providing error bounds of ±10% [13]. Strain gauge-based methods, however, have limitations in the lack of measurement point, difficulty in capturing small variation of the deformation, and errors induced by hole-eccentricity. Due to the limitation of the strain gauge, stress estimation can be insufficiently accurate. 

As an alternative to the strain gauge-based SRM, interferometry techniques are applied to measure small perturbation introduced by drilling a hole offering a full-field deformation measurement [18,19,20,21,22,23]. In general, interferometric pattern-based research works employ holographic pattern, moire pattern, or electronic speckle pattern, of which change reflects deformation induced by drilling a hole. The interferometry-based method provides sensitive deformation measurement that the small size of hole can be utilized. For example, Steinzig and Takahashi [21] estimated residual stress of a 12 mm thick 2024–T351 aluminum ring using 1.59 mm diameter of hole-drilling with 8.5% error. Schmitt et al. [22] used electronic speckle pattern interferometry using 5 mm of hole diameter to obtain the residual stress with the error of 37% for the worst case. Albertazzi et al. [23] conducted a comparative study between strain gauge method and digital speckle pattern interferometry method by measuring stress in a 3 m long carbon steel bar showing 19% of maximum deviation between two methods. As such, the interferometry-based method shows strong potential on the residual stress estimation. Since creating the interferometric pattern is not a simple problem, researchers endeavor to build a compact measurement system for practical applications.

Digital image correlation (DIC) has been introduced to monitor the stresses on full-scale structures [24,25,26,27,28,29] that can replace strain-based and interferometry-based methods. DIC uses two images, one before and one after deformation, by which displacement field is calculated through searching the maximum correlation for each sub-divided pixel groups, also called subsets [30,31]. Since displacement at each pixel is calculated, DIC provides dense displacement field. In the meantime, DIC use a spayed pattern on the specimen without laser beam; hence can replace both the strain-based and the interferometry-based methods offering dense deformation data and simple measurement setup. Trautner et al. [15] used core drilling and DIC to measure the stress level of their specimen. Whereas the stress was successfully estimated with 10% error, the core of 150 mm diameter used in the experiment was considered to be impractical for general civil-engineering structures. Lee et al. [29] also applied the SRM with DIC to measure the stress of concrete specimens, drilling a hole with a diameter of 10 mm and a depth of 40 mm. While the inflicted damage was small, the stress estimation results showed a large variation, with errors from 5.67% to 29.13%. Since DIC generally assumes that a camera takes images at a fixed location, the camera used in the experiment was located 2 m away from the specimen to prepare space for a drill. Thus, the camera resolution of 0.08 mm/pixel at the 2 m distance was not sufficiently high for reliable and consistent stress estimation. Although SRM/DIC-based stress estimation methods show strong potential for field application, drilling-induced camera vibration can be reflected as a DIC error in the displacement field which has not been addressed in the existing literatures.

This study proposes an SRM/DIC-based stress estimation approach considering camera relocation before and after the hole is drilled. The proposed method assumes that the camera is placed close to a specimen for maximum pixel density, removed while the hole is drilled, and placed at the original location to take the after-damage image. Since the camera cannot be placed at the same location, the difference in the camera location is reflected as displacement error in the DIC result. Provided that the displacement measured by DIC is simultaneously induced by stress relaxation and movement of the camera, the proposed method compensates for the component of camera movement. Numerical and experimental validations are conducted to demonstrate the successful removal of the camera motion-induced errors and the resulting stress estimation.

## 2. Residual Stress Estimation

The residual stress can be estimated by using the hole-drilling method in conjunction with the DIC technique. This method, however, exhibits unreliable stress estimation due to the camera movement during the hole-drilling process. This section briefly introduces the basics of the residual stress estimation, and then, a practical limitation of the residual stress method is discussed.

### 2.1. Residual Stress Estimation

**Figure 1:**
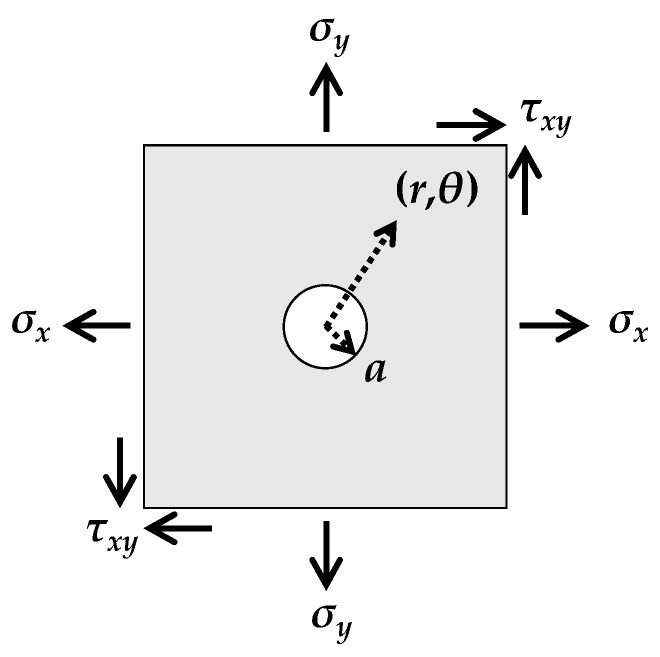
Stress configuration and coordinate system in the hole-drilling method.

Drilling a hole in an isotropic plate results in redistribution of the displacement field, the pattern of which is described well by the Kirsch equation [8,9]. Note that the conventional Kirsch equation deals with an isotropic material under linear elastic behavior, thereby an unexpected error can be observed due to plasticity and anisotropic properties [13,32,33]. Consider an infinite plate under uniform biaxial stresses over the material, as shown in Figure 1. Based on the Kirsch equation, the perturbation introduced by a circular hole is expressed as

(1) $$\left\{\begin{array}{c} u_{hole}(x_{i},y_{i})\\ v_{hole}(x_{i},y_{i}) \end{array}\right\}=\left\{\begin{array}{c} P_{u}(x_{i},y_{i})\\ P_{v}(x_{i},y_{i}) \end{array}\right\}\sigma_{x}+\left\{\begin{array}{c} Q_{u}(x_{i},y_{i})\\ Q_{v}(x_{i},y_{i}) \end{array}\right\}\sigma_{y}+\left\{\begin{array}{c} R_{u}(x_{i},y_{i})\\ R_{v}(x_{i},y_{i}) \end{array}\right\}\tau_{xy},$$

where *u_hole_*(*x_i_*, *y_i_*) and *v_hole_*(*x_i_*, *y_i_*) are, respectively, the *x*- and *y*-directional displacements at the *i*-th material position (*x_i_*, *y_i_*), induced by the residual stresses (*σ_x_*, *σ_y_*, *τ_xy_*). The stress coefficients *P_u_*, *Q_u_*, *R_u_*, *P_v_*, *Q_v_*, and *R_v_* are expanded as

(2) $$P_{u}(x_{i},y_{i})=[A_{i}+(B_{i}+C_{i})\cos(2\theta_{i})-C_{i}]\cos(\theta_{i})$$

(3) $$Q_{u}(x_{i},y_{i})=[A_{i}-B_{i}\cos(2\theta_{i})]\cos(\theta_{i})+C_{i}\sin(2\theta_{i})\sin(\theta_{i}),$$

(4) $$R_{u}(x_{i},y_{i})=+2[(B_{i}+C_{i})\cos(2\theta_{i})+B_{i}]\sin(\theta_{i}),$$

(5) $$P_{v}(x_{i},y_{i})=[A_{i}+(B_{i}+C_{i})\cos(2\theta_{i})+C_{i}]\cos(\theta_{i}),$$

(6) $$Q_{v}(x_{i},y_{i})=[A_{i}-(B_{i}+C_{i})\cos(2\theta_{i})-C_{i}]\sin(\theta_{i}),$$

(7) $$R_{v}(x_{i},y_{i})=-2[(B_{i}+C_{i})\cos(2\theta_{i})-B_{i}]\cos(\theta_{i}),$$

where *θ_i_* is the angular coordinate of the location (*x_i_*, *y_i_*) in the polar coordinate system. The coefficients *A_i_*, *B_i_*, and *C_i_* are expanded as

(8) $$A_{i}=\frac{a^{2}(1+\nu )}{2E}\frac{1}{r_{i}},$$

(9) $$B_{i}=\frac{2a^{2}}{E}\frac{1}{r_{i}}-\frac{a^{4}(1+\nu )}{2E}\frac{1}{r_{i}{}^{3}},$$

(10) $$C_{i}=-\frac{a^{2}(1-\nu )}{E}\frac{1}{r_{i}}--\frac{a^{4}(1+\nu )}{2E}\frac{1}{r_{i}{}^{3}},$$

where *r_i_* is the radial coordinate of the location (*x_i_*, *y_i_*) in the polar coordinate system; *a* is the radius of the hole; *υ* is Poisson’s ratio; and *E* is the Young’s modulus of the material. Using Equation (1), the biaxial residual stresses can be reformulated in the form of a linear regression form as

(11) $$\left\{\begin{array}{c} \sigma_{x}\\ \sigma_{y}\\ \tau_{xy} \end{array}\right\}={\left[\begin{array}{ccc} P_{u}(x_{1},y_{1}) & Q_{u}(x_{1},y_{1}) & R_{u}(x_{1},y_{1})\\ P_{v}(x_{1},y_{1}) & Q_{v}(x_{1},y_{1}) & R_{v}(x_{1},y_{1})\\ \vdots & \vdots & \vdots \\ P_{u}(x_{n},y_{n}) & Q_{u}(x_{n},y_{n}) & R_{u}(x_{n},y_{n})\\ P_{v}(x_{n},y_{n}) & Q_{v}(x_{n},y_{n}) & R_{v}(x_{n},y_{n}) \end{array}\right]}^{+}\left\{\begin{array}{c} u_{hole}(x_{1},y_{1})\\ v_{hole}(x_{1},y_{1})\\ \vdots \\ u_{hole}(x_{n},y_{n})\\ v_{hole}(x_{n},y_{n}) \end{array}\right\},$$

where [·]^+^ is the Moore–Penrose inverse [34]; *n* is the number of locations where displacements *u_hole_* and *v_hole_* are measured; and *P_u_*, *Q_u_*, *R_u_*, *P_v_*, *Q_v_*, and *R_v_* are the stress coefficients calculated at every measurement location. Once the 3 × 2*n* premultiplying matrix in Equation (11) is established based on the foreknown information (*E*, *υ*, *a*, *x_i_*, *y_i_*) for 1 ≤ *i* ≤ *n*, the biaxial residual stresses can be estimated using the displacement field measured by a deformation measurement technique.

**Figure 2:**
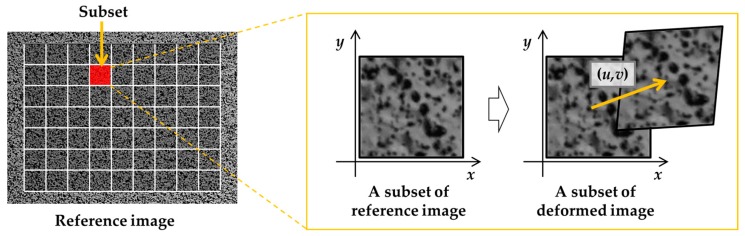
Displacement field calculation using the digital image correlation (DIC) technique.

The full-field displacement required in Equation (11) can be obtained by means of the DIC technique. Images of a specimen, of which surface is prepared with a random speckle pattern by spraying paints, are captured before and after the deformation. Here, the captured images are respectively denoted as the reference and deformed images. The reference image is divided into subsets, as shown in Figure 2. The new position of each of the subsets in the deformed image is searched using a correlation criterion such as cross-correlation, normalized cross-correlation, or zero-normalized cross-correlation [30,31]. Finally, the displacements computed for each subset are collected to construct a two-dimensional displacement field. Therefore, the generic DIC method calculates the displacement field by tracking the position of the material points using a correlation criterion that can be employed in Equation (11).

The overall procedure of the residual stress estimation involves drilling a hole and acquiring images. By drilling a hole, the underlying residual stresses in the target material are relaxed, thus deforming the surface texture around the hole. Using the images before and after the hole is drilled, the DIC technique calculates the deformation field. Finally, the residual stresses in the specimen are estimated based on the linear relationship between the amount of released deformation and the level of relaxed stress, as formulated in Equation (11). Here, improper measurement of the displacement field adversely affects the stress estimation. Thus, for a reliable stress estimation, the camera needs to be fixed at a stationary point during the hole-drilling process. 

### 2.2. Residual Stress Estimation with Camera Motion-Induced Error

**Figure 3:**
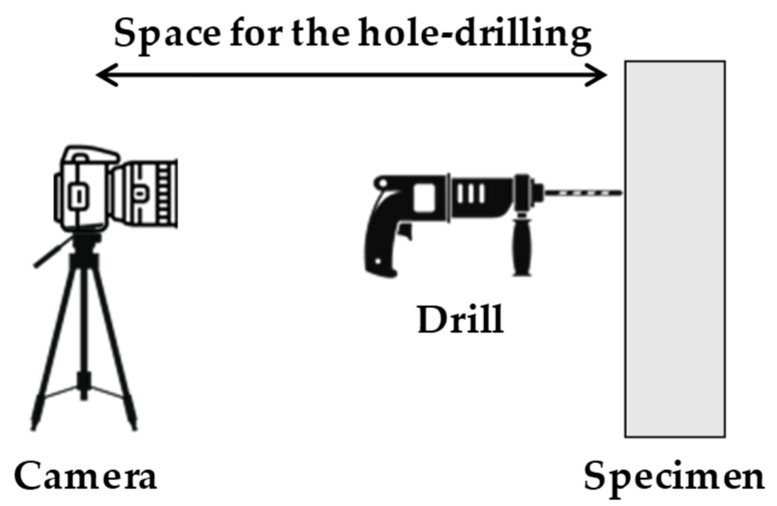
Hardware configuration for the hole-drilling methods integrated with the DIC technique.

Camera motion is inevitable, owing to the hole-drilling process. Consider a common hardware configuration of the residual stress estimation method, as shown in Figure 3, in which the camera needs to be fixed during the hole-drilling process. The process is accompanied by near-field vibration, which causes camera motion. To avoid this vibration, the camera is placed away from the specimen, but this long working distance produces errors, even with a slight vibration of the camera. As such, camera motion is difficult to avoid considering the practical implementation of the hole-drilling method.

**Figure 4:**
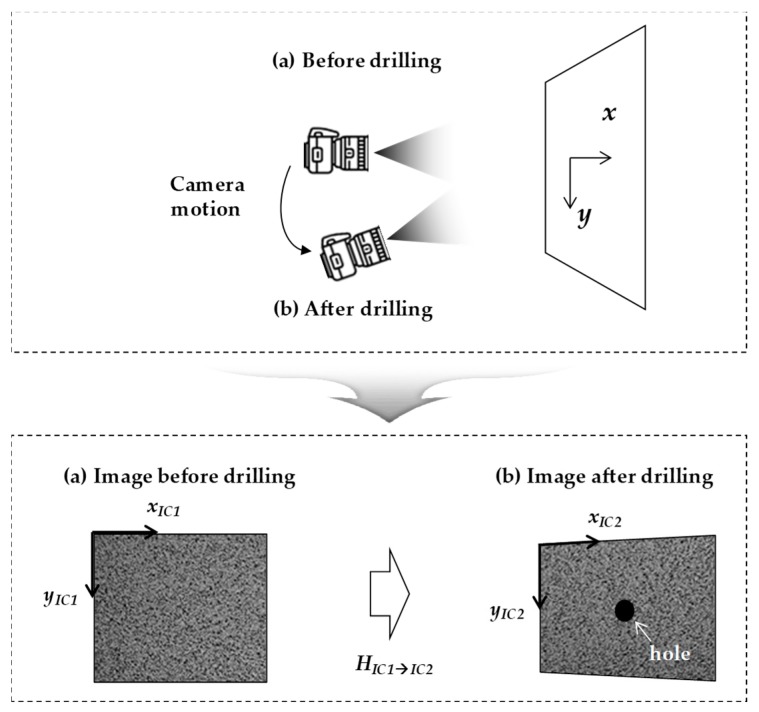
Illustration of the camera motion during hole drilling.

The displacement field induced by the camera motion can be formulated using the homography transform. Let the image coordinate systems before and after the hole-drilling process be respectively denoted as *IC*1 and *IC*2, as shown in Figure 4. The relationship between *IC*1 and *IC*2 is expressed as

(12) $${\left\{\begin{array}{c} x_{i}\\ y_{i}\\ 1 \end{array}\right\}}_{IC2}=sH_{IC1\to IC2}{\left\{\begin{array}{c} x_{i}\\ y_{i}\\ 1 \end{array}\right\}}_{IC1},$$

where {*x_i_*, *y_i_*, 1}^T^*_IC_*_1_ and {*x_i_*, *y_i_*, 1}^T^*_IC_*_2_ are the image coordinates of the *i*-th material point represented in *IC*1 and *IC*2, respectively; *H_IC_*_1__→*IC*2_ is the homography transform associated with the six degrees-of-freedom (6DOF) camera motion [35]; and *s* is the scaling factor that holds the third component of {*x*, *y*, 1}^T^*_IC_*_2_ in 1. Using the homography relationship, the camera motion-induced displacement field is formulated as

(13) $$\left\{\begin{array}{c} u_{motion}(x_{i},y_{i})\\ v_{motion}(x_{i},y_{i}) \end{array}\right\}={\left\{\begin{array}{c} x_{i}\\ y_{i} \end{array}\right\}}_{IC2}-{\left\{\begin{array}{c} x_{i}\\ y_{i} \end{array}\right\}}_{IC1},$$

where *u_motion_*(*x_i_*, *y_i_*) and *v_motion_*(*x_i_*, *y_i_*) are, respectively, the *x*- and *y*-directional displacements of the *i*-th material point, induced by the camera motion. Note that the subtraction between the vectors in different coordinate systems are performed by taking the components. Thus, the homography matrix *H_IC_*_1__→*IC*2_ established by the camera motion governs the displacements of all the material points.

The effect of the camera motion in the residual stress estimation can be simplified based on Equations (1) and (12). The position of the *i*-th material point after hole drilling is expressed as

(14) $${\left\{\begin{array}{c} x_{hole}(x_{i},y_{i})\\ y_{hole}(x_{i},y_{i})\\ 1 \end{array}\right\}}_{IC1}={\left\{\begin{array}{c} x_{i}+u_{hole}(x_{i},y_{i})\\ y_{i}+v_{hole}(x_{i},y_{i})\\ 1 \end{array}\right\}}_{IC1},$$

where *x_hole_*(*x_i_*, *y_i_*) and *y_hole_*(*x_i_*, *y_i_*) are, respectively, the *x* and *y* coordinates of the *i*-th material point after the hole drilling represented in *IC*1. Considering the camera motion, the coordinates in Equation (14) are represented in *IC*2 as

(15) $${\left\{\begin{array}{c} x_{hole}(x_{i},y_{i})\\ y_{hole}(x_{i},y_{i})\\ 1 \end{array}\right\}}_{IC2}=sH_{IC1\to IC2}{\left\{\begin{array}{c} x_{hole}(x_{i},y_{i})\\ y_{hole}(x_{i},y_{i})\\ 1 \end{array}\right\}}_{IC1}.$$

The combination of Equations (13) and (15) results in

(16) $$\left\{\begin{array}{c} u_{hole+motion}(x_{i},y_{i})\\ v_{hole+motion}(x_{i},y_{i}) \end{array}\right\}={\left\{\begin{array}{c} x_{hole}(x_{i},y_{i})\\ y_{hole}(x_{i},y_{i}) \end{array}\right\}}_{IC2}-{\left\{\begin{array}{c} x_{i}\\ y_{i} \end{array}\right\}}_{IC1},$$

where *u_hole_*_+*motion*_(*x_i_*, *y_i_*) and *v_hole_*_+*motion*_(*x_i_*, *y_i_*) are the displacements of the *i*-th material point after hole drilling and the camera motion computed by the DIC technique. Thus, the residual stress estimation fails when the camera moves because of the additional displacement from the camera motion.

## 3. Camera Motion-Independent Residual Stress Estimation

This study proposes a residual stress estimation method that offers camera movement during the hole-drilling process. The displacement field measured using DIC is decomposed into homography-dependent and homography-independent components. Associated with the level of residual stresses, the latter is further used for stress estimation considering the camera movement. This section describes the extraction of the homography-independent displacement field, which is then utilized for the residual stress estimation.

### 3.1. Extraction of Homography-Independent Component

DIC yields full-field displacement data, *u_hole_*_+*motion*_(*x_i_*, *y_i_*) and *v_hole_*_+*motion*_(*x_i_*, *y_i_*), which can be decomposed into homography-dependent and homography-independent components. The displacement field induced by the camera motion can only be included in the homography-dependent terms (see Equations (12) and (13)), whereas hole drilling induces the displacement field consisting of the homography-dependent and homography-independent terms as

(17) $$\left\{\begin{array}{c} u_{hole}(x_{i},y_{i})\\ v_{hole}(x_{i},y_{i}) \end{array}\right\}=\left\{\begin{array}{c} u_{h}(x_{i},y_{i})\\ v_{h}(x_{i},y_{i}) \end{array}\right\}+\left\{\begin{array}{c} u_{\bar{h}}(x_{i},y_{i})\\ v_{\bar{h}}(x_{i},y_{i}) \end{array}\right\}$$

where the subscripts *h* and $\bar{h}$, respectively, denote the homography-dependent and homography-independent components within the displacement field induced by the hole drilling. Note that the homography-dependent term can be replaced with the premultiplication of a principal homography matrix *H_h_* as

(18) $$\left\{\begin{array}{c} x_{i}+u_{h}(x_{i},y_{i})+u_{\bar{h}}(x_{i},y_{i})\\ y_{i}+v_{h}(x_{i},y_{i})+v_{\bar{h}}(x_{i},y_{i})\\ 1 \end{array}\right\}=sH_{h}\left\{\begin{array}{c} x_{i}+u_{\bar{h}}(x_{i},y_{i})\\ y_{i}+v_{\bar{h}}(x_{i},y_{i})\\ 1 \end{array}\right\},$$

Equation (18) is coupled with Equation (15) as

(19) $$\;{\left\{\begin{array}{c} x_{i}+u_{h}(x_{i},y_{i})+u_{\bar{h}}(x_{i},y_{i})\\ y_{i}+v_{h}(x_{i},y_{i})+v_{\bar{h}}(x_{i},y_{i})\\ 1 \end{array}\right\}}_{IC2}=sH_{IC1\to IC2}H_{h}\left\{\begin{array}{c} x_{i}+u_{\bar{h}}(x_{i},y_{i})\\ y_{i}+v_{\bar{h}}(x_{i},y_{i})\\ 1 \end{array}\right\},$$

which is, in turn, expressed as

(20) $$\;\left\{\begin{array}{c} x_{i}+u_{hole+motion}(x_{i},y_{i})\\ y_{i}+v_{hole+motion}(x_{i},y_{i})\\ 1 \end{array}\right\}=sH_{IC1\to IC2}H_{h}\left\{\begin{array}{c} x_{i}+u_{\bar{h}}(x_{i},y_{i})\\ y_{i}+v_{\bar{h}}(x_{i},y_{i})\\ 1 \end{array}\right\}.$$

Equation (20) represents the homography-dependent terms in the homography matrices. On the contrary, the homography-independent terms cannot be expressed in a homography matrix. Thus, the displacement field computed using DIC, which is *u_hole_*_+*motion*_(*x_i_*, *y_i_*) and *v_hole_*_+*motion*_(*x_i_*, *y_i_*), consists of the homography-dependent terms that can be expressed by *H_IC1__→IC2_H_h_* and the homography-independent terms $u_{\bar{h}}$ (*x_i_*, *y_i_*) and $v_{\bar{h}}$ (*x_i_*, *y_i_*).

The homography-independent terms can be extracted using the known information: *u_hole_*_+*motion*_(*x_i_*, *y_i_*), *v_hole_*_+*motion*_(*x_i_*, *y_i_*), *x_i_*, and *y_i_*. A unique homography transform can be found when minimizing the residue defined by

(21) $$residue={\sum_{i=1}^{n}\left|\left\{\begin{array}{c} x_{i}+u_{hole+motion}(x_{i},y_{i})\\ y_{i}+v_{hole+motion}(x_{i},y_{i})\\ 1 \end{array}\right\}-sH\left\{\begin{array}{c} x_{i}\\ y_{i}\\ 1 \end{array}\right\}\right|}_{2}={\sum_{i=1}^{n}\left|\left\{\begin{array}{c} u_{\bar{h}}(x_{i},y_{i})\\ v_{\bar{h}}(x_{i},y_{i})\\ 0 \end{array}\right\}\right|}_{2},$$

where ${\left|\cdot \right|}_{2}$ denotes the 2-norm of a vector. This unique homography transform derived in Equation (21) is indeed *H_IC1__→IC2_H_h_* in Equation (20) because the homography-independent terms, $u_{\bar{h}}$(*x_i_*, *y_i_*) and $v_{\bar{h}}$(*x_i_*, *y_i_*), remained as the residue. Equation (20) can be approximately reorganized by using Equation (21) as

(22) $$\;\left\{\begin{array}{c} x_{i}+u_{hole+motion}(x_{i},y_{i})\\ y_{i}+v_{hole+motion}(x_{i},y_{i})\\ 1 \end{array}\right\}=sH\left\{\begin{array}{c} x_{i}\\ y_{i}\\ 1 \end{array}\right\}+\left\{\begin{array}{c} u_{\bar{h}}(x_{i},y_{i})\\ v_{\bar{h}}(x_{i},y_{i})\\ 0 \end{array}\right\}.$$

By employing the direct linear transform [35], *H* in Equation (22) is computed using the correspondences between {*x_i_* + *u_hole_*_+*motion*_(*x_i_*, *y_i_*), *y_i_* + *v_hole_*_+*motion*_(*x_i_*, *y_i_*)} and {*x_i_*, *y_i_*} for 1 ≤ *i* ≤ *n*. Note that $u_{\bar{h}}$(*x_i_*, *y_i_*) and $v_{\bar{h}}$(*x_i_*, *y_i_*) are excluded in the determination of the homography matrix because these terms are homography-independent. After *H* is determined, the homography-independent terms are extracted through

(23) $$\left\{\begin{array}{c} x_{i}+u_{\bar{h}}(x_{i},y_{i})\\ y_{i}+v_{\bar{h}}(x_{i},y_{i})\\ 1 \end{array}\right\}=sH^{-1}\;\left\{\begin{array}{c} x_{i}+u_{hole+motion}(x_{i},y_{i})\\ y_{i}+v_{hole+motion}(x_{i},y_{i})\\ 1 \end{array}\right\},$$

which is further developed as

(24) $$\left\{\begin{array}{c} u_{\bar{h}}(x_{i},y_{i})\\ v_{\bar{h}}(x_{i},y_{i}) \end{array}\right\}=\left\{\begin{array}{c} x_{i}+u_{\bar{h}}(x_{i},y_{i})\\ y_{i}+v_{\bar{h}}(x_{i},y_{i}) \end{array}\right\}-\left\{\begin{array}{c} x_{i}\\ y_{i} \end{array}\right\}.$$

Hence, the homography-independent terms, $u_{\bar{h}}$(*x_i_*, *y_i_*) and $v_{\bar{h}}$(*x_i_*, *y_i_*) for 1 ≤ *i* ≤ *n*, can be extracted by deriving *H* in Equation (22) and using Equation (24). 

### 3.2. Residual Stress Estimation Using the Homography-Independent Terms

The residual stresses can be estimated by solely employing the homography-independent terms derived in Section 3.1. Equation (1) is reinterpreted in terms of homography dependency as

(25) $$\left\{\begin{array}{c} u_{hole}(x_{i},y_{i})\\ v_{hole}(x_{i},y_{i}) \end{array}\right\}=\left\{\begin{array}{c} P_{uh}(x_{i},y_{i})+P_{u\bar{h}}(x_{i},y_{i})\\ P_{vh}(x_{i},y_{i})+P_{v\bar{h}}(x_{i},y_{i}) \end{array}\right\}\sigma_{x}+\left\{\begin{array}{c} Q_{uh}(x_{i},y_{i})+Q_{u\bar{h}}(x_{i},y_{i})\\ Q_{vh}(x_{i},y_{i})+Q_{v\bar{h}}(x_{i},y_{i}) \end{array}\right\}\sigma_{y}+\left\{\begin{array}{c} R_{uh}(x_{i},y_{i})+R_{u\bar{h}}(x_{i},y_{i})\\ R_{vh}(x_{i},y_{i})+R_{v\bar{h}}(x_{i},y_{i}) \end{array}\right\}\tau_{xy},$$

where the additional subscripts *h* and $\bar{h}$ denote the homography-dependent and homography-independent components, respectively. The homography-independent terms in Equation (25) are expressed as

(26) $$\left\{\begin{array}{c} u_{\bar{h}}(x_{i},y_{i})\\ v_{\bar{h}}(x_{i},y_{i}) \end{array}\right\}=\left\{\begin{array}{c} P_{u\bar{h}}(x_{i},y_{i})\\ P_{v\bar{h}}(x_{i},y_{i}) \end{array}\right\}\sigma_{x}+\left\{\begin{array}{c} Q_{u\bar{h}}(x_{i},y_{i})\\ Q_{v\bar{h}}(x_{i},y_{i}) \end{array}\right\}\sigma_{y}+\left\{\begin{array}{c} R_{u\bar{h}}(x_{i},y_{i})\\ R_{v\bar{h}}(x_{i},y_{i}) \end{array}\right\}\tau_{xy}.$$

The residual stresses are computed as

(27) $$\left\{\begin{array}{c} \sigma_{x}\\ \sigma_{y}\\ \tau_{xy} \end{array}\right\}={\left[\begin{array}{ccc} P_{u\bar{h}}(x_{1},y_{1}) & Q_{u\bar{h}}(x_{1},y_{1}) & R_{u\bar{h}}(x_{1},y_{1})\\ P_{v\bar{h}}(x_{1},y_{1}) & Q_{v\bar{h}}(x_{1},y_{1}) & R_{v\bar{h}}(x_{1},y_{1})\\ \vdots & \vdots & \vdots \\ P_{u\bar{h}}(x_{n},y_{n}) & Q_{u\bar{h}}(x_{n},y_{n}) & R_{u\bar{h}}(x_{n},y_{n})\\ P_{v\bar{h}}(x_{n},y_{n}) & Q_{v\bar{h}}(x_{n},y_{n}) & R_{v\bar{h}}(x_{n},y_{n}) \end{array}\right]}^{+}\left\{\begin{array}{c} u_{\bar{h}}(x_{1},y_{1})\\ v_{\bar{h}}(x_{1},y_{1})\\ \vdots \\ u_{\bar{h}}(x_{n},y_{n})\\ v_{\bar{h}}(x_{n},y_{n}) \end{array}\right\}.$$

Once $P_{u\bar{h}}$, $Q_{u\bar{h}}$, $R_{u\bar{h}}$, $P_{v\bar{h}}$, $Q_{v\bar{h}}$, and $R_{v\bar{h}}$ are calculated for all the material points, the residual stresses can be obtained by using Equation (27) with the homography-independent terms.

The homography-independent coefficients (i.e., $P_{u\overset{¯}{h}}$, $Q_{u\bar{h}}$, $R_{u\bar{h}}$, $P_{v\overset{¯}{h}}$, $Q_{v\bar{h}}$, and $R_{v\bar{h}}$) can be derived by numerically generating displacement fields. Firstly, homography-dependent displacement fields for given in-plane stresses, which is denoted by *u_hole_*(*x_i_*, *y_i_*; *σ_x_*, *σ_y_*, *τ_xy_*) and *v_hole_*(*x_i_*, *y_i_*; *σ_x_*, *σ_y_*, *τ_xy_*), are numerically generated. For given in-plane stresses, *u_hole_*(*x_i_*, *y_i_*) and *v_hole_*(*x_i_*, *y_i_*) can be numerically generated using *E*, *ν*, *a*, *n*, and the pixel location (*x_i_*, *y_i_*) for every pixel based on Equations (1)–(10). Using the correspondences between {*x_i_* + *u_hole_*(*x_i_*, *y_i_*), *y_i_* + *v_hole_*(*x_i_*, *y_i_*)} and {*x_i_*, *y_i_*} for 1 ≤ *i* ≤ *n*, the principal homography transform *H_h_* in Equation (18) can be approximately calculated using direct linear transform [35] with the residue terms as

(28) $$residue={\sum_{i=1}^{n}\left|\left\{\begin{array}{c} x_{i}+u_{hole}(x_{i},y_{i};\sigma_{x},\sigma_{y},\tau_{xy})\\ y_{i}+v_{hole}(x_{i},y_{i};\sigma_{x},\sigma_{y},\tau_{xy})\\ 1 \end{array}\right\}-sH_{h}\left\{\begin{array}{c} x_{i}\\ y_{i}\\ 1 \end{array}\right\}\right|}_{2}={\sum_{i=1}^{n}\left|\left\{\begin{array}{c} u_{\bar{h}}(x_{i},y_{i};\sigma_{x},\sigma_{y},\tau_{xy})\\ v_{\bar{h}}(x_{i},y_{i};\sigma_{x},\sigma_{y},\tau_{xy})\\ 0 \end{array}\right\}\right|}_{2}.$$

Using the calculated *H_h_*, Equation (18) can be reorganized as

(29) $$\left\{\begin{array}{c} x_{i}+u_{h}(x_{i},y_{i};\sigma_{x},\sigma_{y},\tau_{xy})\\ y_{i}+v_{h}(x_{i},y_{i};\sigma_{x},\sigma_{y},\tau_{xy})\\ 1 \end{array}\right\}=sH_{h}\left\{\begin{array}{c} x_{i}\\ y_{i}\\ 1 \end{array}\right\}.$$

As a result, *u_hole_*(*x_i_*, *y_i_*; *σ_x_*, *σ_y_*, *τ_xy_*) and *v_hole_*(*x_i_*, *y_i_*; *σ_x_*, *σ_y_*, *τ_xy_*) can be prepared for any given stress combination. Second step is to find the homography-independent terms $P_{u\bar{h}}$, $Q_{u\bar{h}}$, $R_{u\bar{h}}$, $P_{v\bar{h}}$, $Q_{v\bar{h}}$, and $R_{v\bar{h}}$ using a number of numerically generated *u_hole_*(*x_i_*, *y_i_*; *σ_x_*, *σ_y_*, *τ_xy_*) and *v_hole_*(*x_i_*, *y_i_*; *σ_x_*, *σ_y_*, *τ_xy_*). For computational simplicity, assume that the homography-dependent terms $P_{uh}$, $Q_{uh}$, $R_{uh}$, $P_{vh}$, $Q_{vh}$, and $R_{vh}$ are linearized as

(30) $$\left\{\begin{array}{c} u_{h}(x_{i},y_{i})\\ v_{h}(x_{i},y_{i}) \end{array}\right\}=\left[\begin{array}{c} P_{u\bar{h},x}x_{i}+P_{u\bar{h},y}y_{i}\\ P_{v\bar{h},x}x_{i}+P_{v\bar{h},y}y_{i} \end{array}\right]\sigma_{x}+\left[\begin{array}{c} Q_{u\bar{h},x}x_{i}+Q_{u\bar{h},y}y_{i}\\ Q_{v\bar{h},x}x_{i}+Q_{v\bar{h},y}y_{i} \end{array}\right]\sigma_{y}+\left[\begin{array}{c} R_{u\bar{h},x}x_{i}+R_{u\bar{h},y}y_{i}\\ R_{v\bar{h},x}x_{i}+R_{v\bar{h},y}y_{i} \end{array}\right]\tau_{xy},$$

where $P_{u\bar{h},x}$, $Q_{u\bar{h},x}$, $R_{u\bar{h},x}$, $P_{v\bar{h},x}$, $Q_{v\bar{h},x}$, $R_{v\bar{h},x}$, $P_{u\bar{h},y}$, $Q_{u\bar{h},y}$, $R_{u\bar{h},y}$, $P_{v\bar{h},y}$, $Q_{v\bar{h},y}$, and $R_{v\bar{h},y}$ are constants to be determined. Equation (30) is rearranged as:

(31) $$\left\{\begin{array}{c} P_{u\bar{h},x}\\ P_{u\bar{h},y}\\ Q_{u\bar{h},x}\\ Q_{u\bar{h},y}\\ R_{u\bar{h},x}\\ R_{u\bar{h},y} \end{array}\right\}={\left[\begin{array}{cccccc} x_{1}\sigma_{x} & y_{1}\sigma_{x} & x_{1}\sigma_{y} & y_{1}\sigma_{x} & x_{1}\tau_{xy} & y_{1}\tau_{xy}\\ \vdots & \vdots & \vdots & \vdots & \vdots & \vdots \\ x_{n}\sigma_{x} & y_{n}\sigma_{x} & x_{n}\sigma_{y} & y_{n}\sigma_{x} & x_{n}\tau_{xy} & y_{n}\tau_{xy} \end{array}\right]}^{+}\left\{\begin{array}{c} u_{h}(x_{1},y_{1})\\ \vdots \\ u_{h}(x_{n},y_{n}) \end{array}\right\}$$

and

(32) $$\left\{\begin{array}{c} P_{v\bar{h},x}\\ P_{v\bar{h},y}\\ Q_{v\bar{h},x}\\ Q_{v\bar{h},y}\\ R_{v\bar{h},x}\\ R_{v\bar{h},y} \end{array}\right\}={\left[\begin{array}{cccccc} x_{1}\sigma_{x} & y_{1}\sigma_{x} & x_{1}\sigma_{y} & y_{1}\sigma_{x} & x_{1}\tau_{xy} & y_{1}\tau_{xy}\\ \vdots & \vdots & \vdots & \vdots & \vdots & \vdots \\ x_{n}\sigma_{x} & y_{n}\sigma_{x} & x_{n}\sigma_{y} & y_{n}\sigma_{x} & x_{n}\tau_{xy} & y_{n}\tau_{xy} \end{array}\right]}^{+}\left\{\begin{array}{c} v_{h}(x_{1},y_{1})\\ \vdots \\ v_{h}(x_{n},y_{n}) \end{array}\right\}.$$

Since the homography-dependent terms *u_h_*(*x_i_*, *y_i_*) and *v_h_*(*x_i_*, *y_i_*) for 1 ≤ *i* ≤ *n* are numerically computed using Equation (29), the unknown constants ($P_{u\bar{h},x}$, $Q_{u\bar{h},x}$, $R_{u\bar{h},x}$, $P_{v\bar{h},x}$, $Q_{v\bar{h},x}$, $R_{v\bar{h},x}$, $P_{u\bar{h},y}$, $Q_{u\bar{h},y}$, $R_{u\bar{h},y}$, $P_{v\bar{h},y}$, $Q_{v\bar{h},y}$, and $R_{v\bar{h},y}$) can be calculated using Equations (31) and (32) with known (*x_i_*, *y_i_*) and (*σ_x_*, *σ_y_*, *τ_xy_*). Once the unknown constants are calculated, $P_{u\bar{h}}$, $Q_{u\bar{h}}$, $R_{u\bar{h}}$, $P_{v\bar{h}}$, $Q_{v\bar{h}}$, and $R_{v\bar{h}}$ can be obtained using Equation (25), and in turn, the residual stresses can be calculated by using Equation (27) with the homography-independent terms $u_{\bar{h}}$(*x_i_*, *y_i_*) and $v_{\bar{h}}$(*x_i_*, *y_i_*) for 1 ≤ *i* ≤ *n*. 

### 3.3. Summary of the Proposed Method

The procedure of the proposed stress estimation, compensating for the 6DOF camera motions, is summarized as follows:

1. *E*, *ν*, *a*, *n*, *x*, and *y* are given before the measurement based on the material properties, hole-drilling scheme, and image resolution.
2. Derive $P_{u}$, $Q_{u}$, $R_{u}$, $P_{v}$, $Q_{v}$, and $R_{v}$ using the given *E*, *ν*, *a*, *n*, *x*, and *y* (known at step 1) based on Equation (2)–(10).
3. Derive $P_{uh}$, $Q_{uh}$, $R_{uh}$, $P_{vh}$, $Q_{vh}$, and $R_{vh}$ by numerically generating multiple pairs of displacement fields (*u_h_*, *v_h_*) and residual stresses (*σ_x_*, *σ_y_*, *τ_xy_*) based on Equations (31) and (32).
4. Derive $P_{u\bar{h}}$, $Q_{u\bar{h}}$, $R_{u\bar{h}}$, $P_{v\bar{h}}$, $Q_{v\bar{h}}$, and $R_{v\bar{h}}$ by subtracting $P_{uh}$, $Q_{uh}$, $R_{uh}$, $P_{vh}$, $Q_{vh}$, and $R_{vh}$ (derived at step 3), respectively from $P_{u}$, $Q_{u}$, $R_{u}$,$\;P_{v}$, $Q_{v}$, and $R_{v}$ (derived at step 2) based on Equation (25).
5. Obtain the displacement field *u_hole+motion_* and *v_hole+motion_* by applying DIC to the images of before and after the hole drill engaged with camera motion.
6. Compute *H* in Equation (22) by using correspondences between {*x*, *y*} (known at step 1) and {*u_hole+motion_*, *v_hole+motion_*} (obtained at step 5) for all material points using direct linear transform [35].
7. Compute $u_{\bar{h}}$ and $v_{\bar{h}}$ by using *u_hole+motion_* and *v_hole+motion_* (obtained at step 5) in combination with *H* (obtained at step 6) based on Equations (22)–(24).
8. Compute the residual stresses *σ_x_*, *σ_y_*, and *τ_xy_* using $u_{\bar{h}}$ and $v_{\bar{h}}$ (obtained at step 7) in combination with $P_{u\bar{h}}$, $Q_{u\bar{h}}$, $R_{u\bar{h}}$, $P_{v\bar{h}}$, $Q_{v\bar{h}}$, and $R_{v\bar{h}}$ (derived at step 4) using Equation (27).

**Figure 5:**
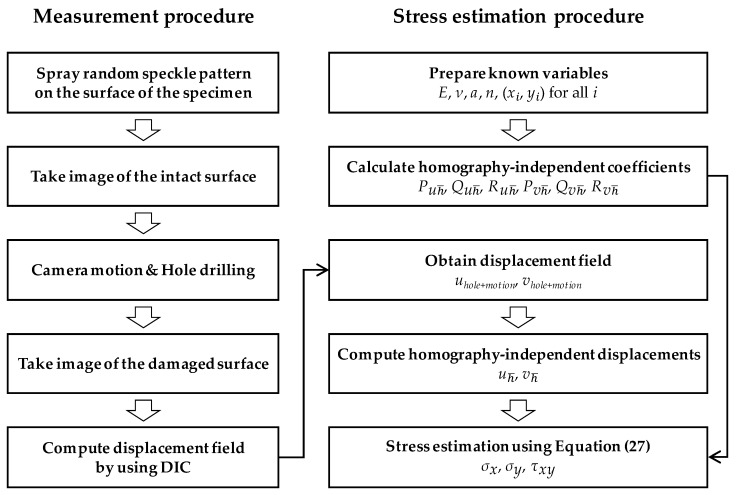
Flowchart of the proposed stress estimation.

The overall proposed SRM/DIC method consists of displacement field measurement and stress estimation, as shown in Figure 5. 

## 4. Numerical Validation

To validate the proposed method, the stress estimation involving hole drilling and camera motion is numerically simulated. For any in-plane stresses and any 6DOF camera motions, the displacement fields *u_hole+motion_* and *v_hole+motion_*, which are employed in the proposed residual stress estimation, are numerically generated. The stresses estimated using the proposed method are compared with the actual stresses applied. This section demonstrates the numerical generation of the displacement fields under movement of the camera before and after the hole is drilled, and presents a comparison between the estimated and actual stresses.

### 4.1. Numerical Generation of the Displacement Field

**Figure 6:**
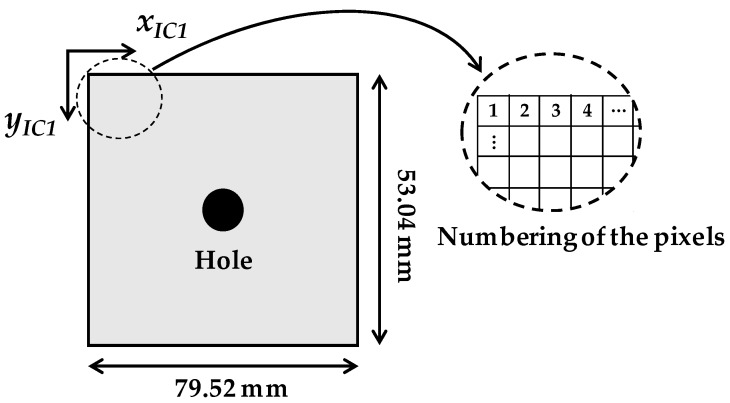
Testing environment for the numerical simulation.

The displacement field induced by drilling a hole is numerically generated for a commercial grade camera system. The camera, equipped with a macro lens and having the specifications listed in Table 1, is numerically modeled to have 79.52 × 53.04 mm of field of view, which can be achieved by adjusting the working distance to 100 mm. Each pixel in the image of 7952 × 5304 resolution is indexed such that (*x_i_*, *y_i_*) for every *i*-th pixel is appropriately allocated, as shown in Figure 6. The Young’s modulus (*E*), Poisson’s ratio (*ν*), and diameter of the hole (*a*) are assumed to be 206 GPa, 0.3, and 12 mm, respectively. Hence, given the in-plane stresses (i.e., *σ_x_*, *σ_y_*, *τ_xy_*), the displacement field, *u_hole_*(*x_i_*, *y_i_*) and *v_hole_*(*x_i_*, *y_i_*), can be established using Equation (1) with the known variables *E*, *ν*, and *a*.

The stress-related terms *u_hole_*(*x_i_*, *y_i_*) and *v_hole_*(*x_i_*, *y_i_*) are affected by the 6DOF camera motion, which affects the final displacement field data *u_hole+motion_*(*x_i_*, *y_i_*) and *v_hole+motion_*(*x_i_*, *y_i_*). The camera mentioned in Table 1 is modeled such that the intrinsic parameter *K* becomes

(33) $$K=\left[\begin{array}{cccc} 10000 & 0 & 3976 & 0\\ 0 & 10000 & 2652 & 0\\ 0 & 0 & 1 & 0 \end{array}\right].$$

Assuming that the initial working distance is 100 mm, the coordinate transformation from the target surface to *IC*1 can be represented as

(34) $$K_{1}=K\left[\begin{array}{cccc} 1 & 0 & 0 & 0\\ 0 & 1 & 0 & 0\\ 0 & 0 & 1 & 100\\ 0 & 0 & 0 & 1 \end{array}\right].$$

The coordinate transformation from the target surface toward *IC*2 is represented as

(35) $$K_{2}=K\left[\begin{array}{cc} R & T\\ 0^{Τ} & 1 \end{array}\right]\left[\begin{array}{cccc} 1 & 0 & 0 & 0\\ 0 & 1 & 0 & 0\\ 0 & 0 & 1 & 100\\ 0 & 0 & 0 & 1 \end{array}\right],$$

where *R*, *T*, and 0^T^ are respectively a 3 × 3 rotation matrix, a 3 × 1 translation vector, and a 1 × 3 zero vector. The homography matrix *H_IC_*_1__→*IC*2_ in Equation (12) is calculated as

(36) $$H_{IC1\to IC2}={\left[\begin{array}{ccc} K_{2,1} & K_{2,2} & K_{2,4} \end{array}\right]}^{-1}\left[\begin{array}{ccc} K_{1,1} & K_{1,2} & K_{1,4} \end{array}\right],$$

where *K*_1,*j*_ and *K*_2,*j*_ are the *j*-th column of *K*_1_ and *K*_2_, respectively. For any given camera motion *R* and *T*, *H_IC_*_1__→*IC*2_ can be constructed for the camera model mentioned in Table 1. Consequently, using *u_hole_*(*x_i_*, *y_i_*), *v_hole_*(*x_i_*, *y_i_*), and *H_IC_*_1__→*IC*2_ for any given in-plane stresses and camera motions, the displacement field, *u_hole+motion_*(*x_i_*, *y_i_*) and *v_hole+motion_*(*x_i_*, *y_i_*), can constructed based on Equations (14)–(16). 

### 4.2. Stress Estimation Using the Proposed Method

To evaluate the performance of the proposed method, the displacement fields are constructed for various stress conditions and camera motions, as listed in Table 2. Herein, *r_x_*, *r_y_*, and *r_z_* are, respectively, the rotation about the *x*, *y*, and *z* axes, which comprise the rotation matrix *R*. In addition, *t_x_*, *t_y_*, and *t_z_* are, respectively, the translations along the *x*, *y*, and *z* axes, which comprise the translation vector *T*. A total of 100 test cases are considered. From Test 1 to Test 60, the camera motions with rotation and translation for a specific stress combination (i.e., *σ_x_* = 20 MPa, *σ_y_* = −5 MPa, and *τ_xy_* = 2 MPa) are considered. From Test 61 to Test 100, the 6DOF camera motions are controlled such that the applied single-DOF motions are increasingly applied from −10 to 10 mrad for the rotations and from −5 to 5 mm for the translations. For Tests 61–100, random combinations of stresses and camera motions are considered, in which *unif*(α, β) is the randomly selected variable from the uniform distribution bounded by (α, β).

**Figure 7:**
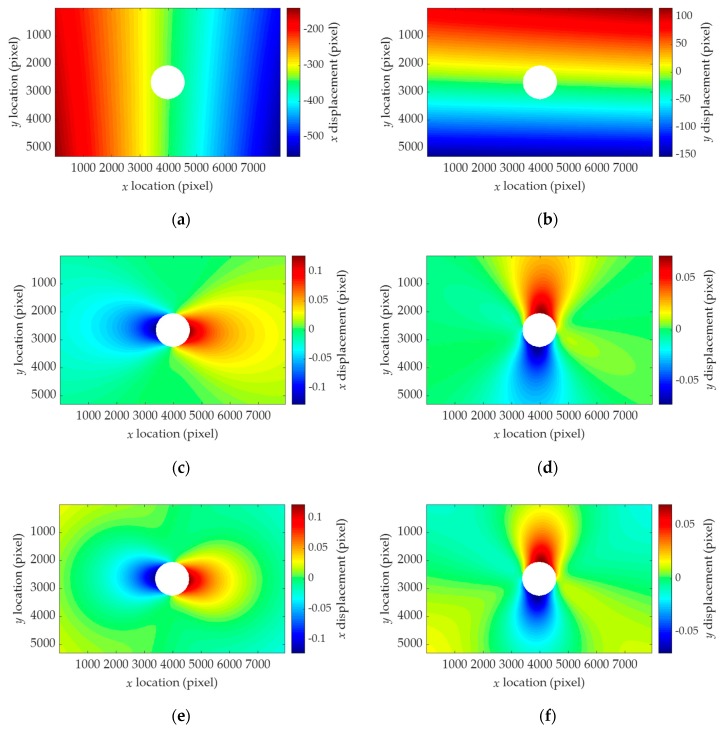
Example of the numerically generated displacement fields for (*σ_x_*, *σ_y_*, *τ_xy_*) = (20, −5, 2) MPa, (*r_x_*, *r_y_*, *r_z_*) = (3, −5, 5) mrad, and (*t_x_*, *t_y_*, *t_z_*) = (3, −2, −5) mm: (**a**) *u_hole+motion_*; (**b**) *v_hole+motion_*; (**c**) *u_hole_*; (**d**) *v_hole_*; (**e**) $u_{\bar{h}}$; (**f**) $v_{\bar{h}}$.

To estimate the residual stresses, $u_{\bar{h}}$ and $v_{\bar{h}}$ are extracted from the numerically simulated displacement field *u_hole+motion_* and *v_hole+motion_*. As can be seen in Figure 7a,b, the displacement fields are primarily dominated by the camera motion-induced patterns, which veil those generated by the stress relaxation shown in Figure 7c,d. Note that the stresses can be computed using *u_hole_* and *v_hole_*, as discussed in Section 2.1, whereas the displacement fields cannot be directly obtained owing to the camera motion, as discussed in Section 2.2. The homography-independent term is extracted, as shown in Figure 7e,f, using the direct linear transform, as discussed in Section 3.1. The extracted $u_{\bar{h}}$ and $v_{\bar{h}}$ will be employed in stress estimation.

**Figure 8:**
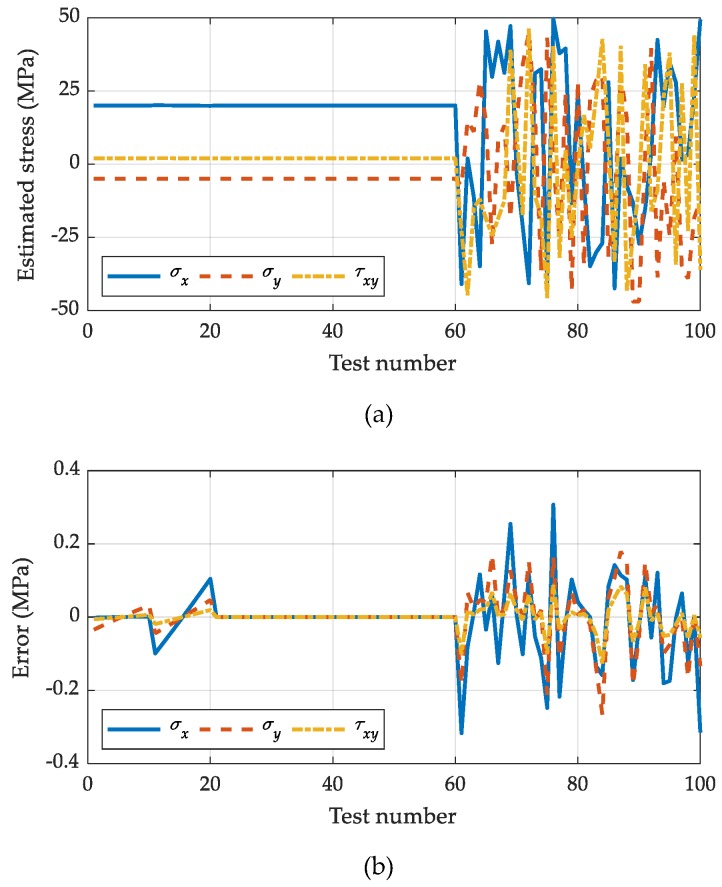
Numerical simulation results for the stress estimation: (**a**) Estimated stresses; (**b**) estimation error.

The stresses are estimated for the scenarios listed in Table 2, using the extracted homography-independent displacement fields $u_{\bar{h}}$ and $v_{\bar{h}}$ . Based on Equation (27), the stresses can be directly obtained by using the $u_{\bar{h}}$ and $v_{\bar{h}}$ calculated for every (*x_i_*, *y_i_*). As exhibited in Figure 8, the stresses estimated by the proposed method agree well with the applied stress. The maximum estimation error is 0.3072 MPa, which is 0.63% of the true stress. Therefore, the proposed method can successfully estimate the residual stresses, even with the camera relocation during the hole-drilling process.

## 5. Laboratory-Scale Validation

**Figure 9:**
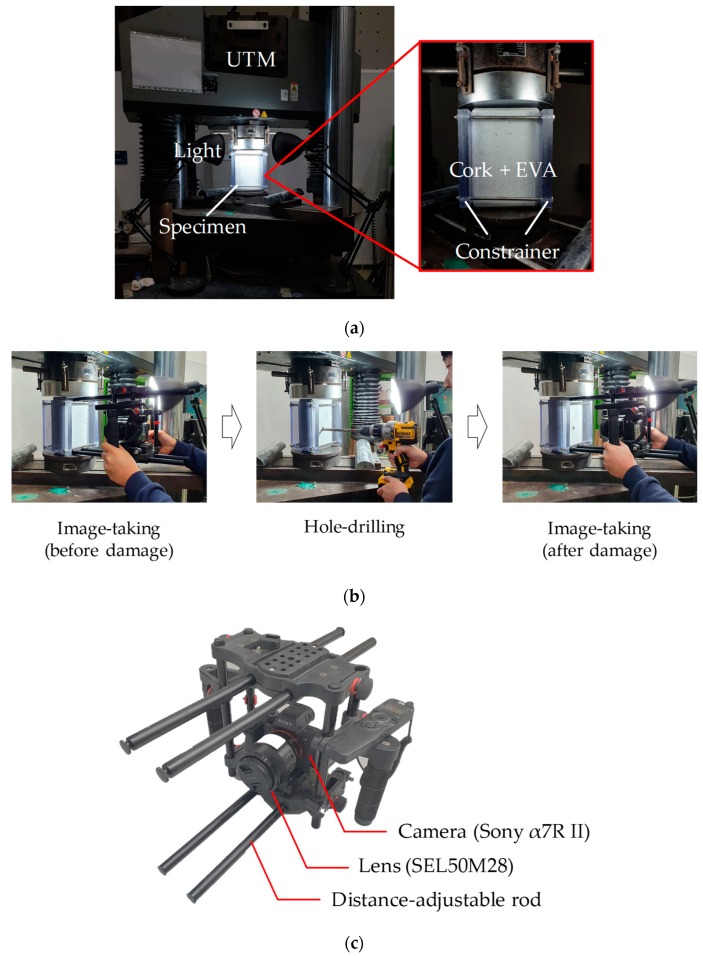
Laboratory-scale experiment: (**a**) Experimental setup; (**b**) experimental procedure; (**c**) camera system.

The proposed method is validated through practical implementations of the stress estimation in a laboratory environment. Three specimens made of cork and ethylene-vinyl acetate (EVA) are prepared, the material properties and dimensions of which are summarized in Table 3. The specimen is compressed using a universal testing machine (UTM), as shown in Figure 9a. Herein, to obtain a two-dimensional stress condition, the horizontal expansion is constrained using polycarbonate plates connected by steel rods. After applying 100 KPa of vertical compression, the image-capturing and hole-drilling processes are performed as depicted in Figure 9b, using the camera system illustrated in Figure 9c. Note that the camera is completely detached and reattached before and after the hole-drilling process, which causes a subtle camera movement. The experiments are repeated with three specimens, and thereby, the images before and after hole drilling are prepared for the post processing.

**Figure 10:**
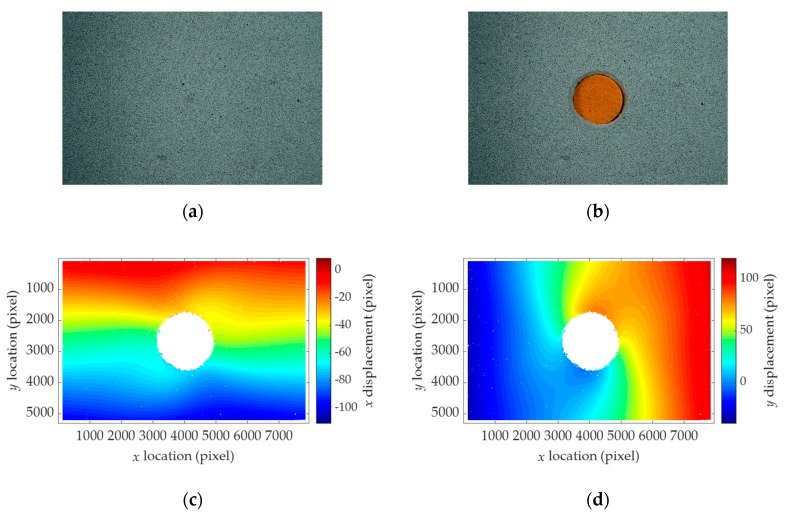
Results of the digital image correlation for Test 3: (**a**) Image of the intact surface; (**b**) image of the drilled surface. An earplug in orange color is placed inside the hole to aid auto focusing; (**c**) *u_hole+motion_*; (**d**) *v_hole+motion_*.

The displacement fields are calculated by using the images before and after the hole-drilling process. Although the images before and after the hole drilling look very similar, as can be seen in Figure 10a,b, the camera motion is certainly present. Ncorr [36], an open-source DIC software, is used for the computation of the displacement fields. A circular subset with a radius of 10 pixels is empirically selected. The resulting displacement fields are shown in Figure 10c,d, in which the camera motion-induced error is dominant. These DIC results are employed in the proposed stress estimation method.

**Figure 11:**
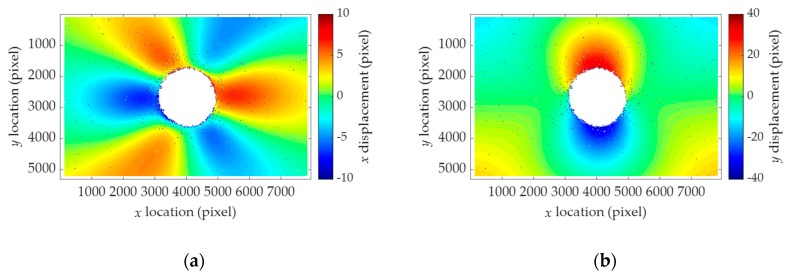
Results of the homography-independent terms for Test 3: (**a**) $u_{\bar{h}}$; (**b**) $v_{\bar{h}}$.

The residual stresses are computed by using the displacement fields. The homography-independent terms are extracted as shown in Figure 11, and are then used to calculate the residual stresses. The estimated stresses are compared with the stress applied by the UTM. As shown in Table 4, the maximum error is calculated to be 7.1%. Even though the error in the numerical simulation was within 0.7%, the laboratory tests result in 1.3% to 7.1% of error due to experimental conditions such as quality of the hole drilling. Nevertheless, 7.1% of error can be considered to be reasonable as previous studies have reported ± 10% of error [13]. Thus, the proposed method reliably measures the residual stresses by successfully compensating the error induced by the camera motion through linear regression using a large amount of displacement field data as discussed previously.

**Figure 12:**
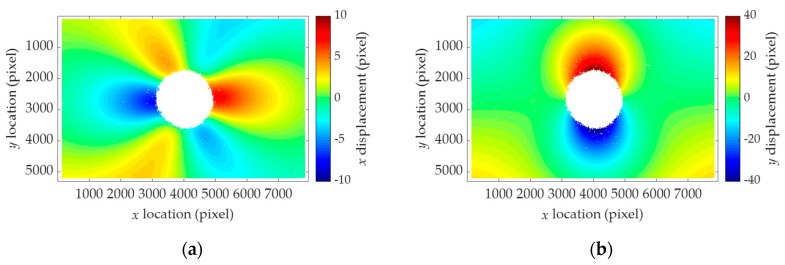
Homography-independent displacement fields reconstructed using the estimated stresses for Test 3: (**a**) Reconstructed $u_{\bar{h}}$; (**b**) reconstructed $v_{\bar{h}}$.

As the hole drilling is a source of measurement error, the goodness of fit is checked after the stress estimation. The hole drilling generally produces a hole with an elliptical shape rather than a perfect circular hole. If the specimen is under compression, the material inside the hole pushes the rotating drill bit during the hole-drilling process because of displacement toward the center of the hole. On the other hand, the tensile zone tends to deviate from the hole center resulting in small damage along the tensile region. As a result, the shape of the hole tends to be an ellipse, which can cause stress estimation error. To investigate the effect of the hole drilling on the measurement accuracy, the goodness of fit is considered. The homography-independent displacement fields are reconstructed using the estimated stresses and Equation (26) as shown in Figure 12. The measured (shown in Figure 11) and the reconstructed displacement fields (shown in Figure 12) exhibit the similar patterns, while the displacement amplitudes are slightly different. To quantitatively evaluate the difference, the root mean square error (RMSE) between the two displacement fields defined in Equation (37) is calculated.

(37) $$RMSE=\frac{1}{n}\sum_{i=1}^{n}\sqrt{{\left(u_{measured}(x_{i},y_{i})-u_{reconstructed}(x_{i},y_{i})\right)}^{2}+{\left(v_{measured}(x_{i},y_{i})-v_{reconstructed}(x_{i},y_{i})\right)}^{2}}$$

where subscripts measured and reconstructed denote measurement and reconstructed displacement fields, respectively. The RMSE can identify mean errors for each pixel. The RMSE values for Tests 1–3 are 1.89 × 10^−4^, 3.41 × 10^−4^, and 3.98 × 10^−4^ pixel, respectively. These values are less than 0.02 pixel of the common DIC error criteria. Thus, the DIC is properly applied with reasonable error levels. 

## 6. Conclusions

This paper proposed an SRM/DIC-based stress estimation method that allows camera relocation for high-resolution image acquisition. Conventionally, DIC uses two images, one before and one after deformation, taken by a fixed camera. As SRM requires hole drilling, the camera needs to be placed away from the specimen to allow space for the drill. To maximize the pixel resolution of the camera, this study developed a stress estimation method in which the camera can be installed directly in front of the specimen. Thus, in the proposed method, the camera is removed while drilling a hole and replaced for taking the after-deformation image. The mathematical formulation for compensation of the camera relocation-induced error was provided in detail. A numerical simulation was conducted for a total of 100 test cases with various camera movements and stress levels, resulting in a maximum error of 0.63%. This study also conducted a laboratory experiment using an EVA specimen. A UTM was used to provide the compressive force that induced 100 KPa of stress in the specimen. The stress levels were estimated with a maximum error of 7.1%. Therefore, the numerical simulation and experiment have shown that the proposed method can accurately measure the stress level, even when the camera is relocated.

## Figures and Tables

**Table 1:** Hardware specification.

Item	Model	Specification
Camera	Sony α7R II	Resolution: 7952 × 5304 pixels
Lens	SEL50M28	Focal length: 50 mm

**Table 2:** Numerical simulation scenario.

Test	Stress (MPa)			Camera Motions					
				Rotation (mrad)			Translation (mm)		
	*σ_x_*	*σ_y_*	*τ_xy_*	*r_x_*	*r_y_*	*r_z_*	*t_x_*	*t_y_*	*t_z_*
1–10	20	−5	2	[−10, 10]	0	0	0	0	0
11–20				0	[−10, 10]	0	0	0	0
21–30				0	0	[−10, 10]	0	0	0
31–40				0	0	0	[−5, 5]	0	0
41–50				0	0	0	0	[−5, 5]	0
51–60				0	0	0	0	0	[−5, 5]
61–100	unif(−50, 50) *			unif(−10, 10) *			unif(−5, 5) *		

* *unif*(α, β) denotes a number randomly selected by the uniform distribution bounded by (α, β).

**Table 3:** Specimen and experimental information.

Property	Value
Young’s modulus	2.46 MPa
Poisson’s ratio	0.1
Dimension (height × width × depth)	230 × 150 × 75 mm
Hole diameter	14 mm
Pixel resolution	7952 × 5304
Pixel density	108.26 pixel/mm

**Table 4:** Experimental results.

Test	True stress (KPa)	Estimated Stresses (KPa)			Error (KPa) *σ_y_*−*σ_y,true_*
	*σ_y,true_*	*σ_x_*	*σ_y_*	*τ_xy_*	
1	−100.0	−29.3	−101.3	+1.2	−1.3
2	−100.0	−21.6	−103.1	−3.7	−3.1
3	−100.0	−39.2	−107.1	−3.0	−7.1

## Nomenclature

*σ_x_*	In-plane residual stress (normal stress along *x* direction).
*σ_y_*	In-plane residual stress (normal stress along *y* direction).
*τ_xy_*	In-plane residual stress (shear stress).
*x_i_*	*x* coordinate of *i*-th material point.
*y_i_*	*y* coordinate of *i*-th material point.
*u_hole_*(*x_i_*, *y_i_*)	*x* directional displacement at {*x_i_*, *y_i_*} induced by hole-drilling.
*v_hole_*(*x_i_*, *y_i_*)	*y* directional displacement at {*x_i_*, *y_i_*} induced by hole-drilling.
*u_motion_*(*x_i_*, *y_i_*)	*x* directional displacement at {*x_i_*, *y_i_*} induced by camera motion.
*v_motion_*(*x_i_*, *y_i_*)	*y* directional displacement at {*x_i_*, *y_i_*} induced by camera motion.
*u_hole+motion_*(*x_i_*, *y_i_*)	*x* directional displacement at {*x_i_*, *y_i_*} induced by hole-drilling and camera motion.
*v_hole+motion_*(*x_i_*, *y_i_*)	*y* directional displacement at {*x_i_*, *y_i_*} induced by hole-drilling and camera motion.
*u_h_*(*x_i_*, *y_i_*)	Homography-dependent *x* directional displacement among *u_hole+motion_*(*x_i_*, *y_i_*).
*v_h_*(*x_i_*, *y_i_*)	Homography-dependent *y* directional displacement among *v_hole+motion_*(*x_i_*, *y_i_*).
$u_{\bar{h}}$(*x_i_*, *y_i_*)	Homography-independent *x* directional displacement among *u_hole+motion_*(*x_i_*, *y_i_*).
$v_{\bar{h}}$(*x_i_*, *y_i_*)	Homography-independent *y* directional displacement among *v_hole+motion_*(*x_i_*, *y_i_*).
*P_u_*	Stress coefficient for linear relationship between *σ_x_* and *u_hole_*(*x_i_*, *y_i_*).
*Q_u_*	Stress coefficient for linear relationship between *σ_y_* and *u_hole_*(*x_i_*, *y_i_*).
*R_u_*	Stress coefficient for linear relationship between *τ_xy_* and *u_hole_*(*x_i_*, *y_i_*).
*P_v_*	Stress coefficient for linear relationship between *σ_x_* and *v_hole_*(*x_i_*, *y_i_*).
*Q_v_*	Stress coefficient for linear relationship between *σ_y_* and *v_hole_*(*x_i_*, *y_i_*).
*R_v_*	Stress coefficient for linear relationship between *τ_xy_* and *v_hole_*(*x_i_*, *y_i_*).
*P_uh_*	Stress coefficient for linear relationship between *σ_x_* and *u_h_*(*x_i_*, *y_i_*).
*Q_uh_*	Stress coefficient for linear relationship between *σ_y_* and *u_h_*(*x_i_*, *y_i_*).
*R_uh_*	Stress coefficient for linear relationship between *τ_xy_* and *u_h_*(*x_i_*, *y_i_*).
*P_vh_*	Stress coefficient for linear relationship between *σ_x_* and *v_h_*(*x_i_*, *y_i_*).
*Q_vh_*	Stress coefficient for linear relationship between *σ_y_* and *v_h_*(*x_i_*, *y_i_*).
*R_vh_*	Stress coefficient for linear relationship between *τ_xy_* and *v_h_*(*x_i_*, *y_i_*).
*P_uh_*	Stress coefficient for linear relationship between *σ_x_* and *u_h_*(*x_i_*, *y_i_*).
*Q_uh_*	Stress coefficient for linear relationship between *σ_y_* and *u_h_*(*x_i_*, *y_i_*).
*R_uh_*	Stress coefficient for linear relationship between *τ_xy_* and *u_h_*(*x_i_*, *y_i_*).
*P_vh_*	Stress coefficient for linear relationship between *σ_x_* and *v_h_*(*x_i_*, *y_i_*).
*Q_vh_*	Stress coefficient for linear relationship between *σ_y_* and *v_h_*(*x_i_*, *y_i_*).
*R_vh_*	Stress coefficient for linear relationship between *τ_xy_* and *v_h_*(*x_i_*, *y_i_*).
$P_{u\bar{h}}$	Stress coefficient for linear relationship between *σ_x_* and $u_{\bar{h}}$(*x_i_*, *y_i_*).
$Q_{u\bar{h}}$	Stress coefficient for linear relationship between *σ_y_* and $u_{\bar{h}}$(*x_i_*, *y_i_*).
$R_{u\bar{h}}$	Stress coefficient for linear relationship between *τ_xy_* and $u_{\bar{h}}$(*x_i_*, *y_i_*).
$P_{v\bar{h}}$	Stress coefficient for linear relationship between *σ_x_* and $v_{\bar{h}}$(*x_i_*, *y_i_*).
$Q_{v\bar{h}}$	Stress coefficient for linear relationship between *σ_y_* and $v_{\bar{h}}$(*x_i_*, *y_i_*).
$R_{v\bar{h}}$	Stress coefficient for linear relationship between *τ_xy_* and $v_{\bar{h}}$(*x_i_*, *y_i_*).
*IC*1	Image coordinate system before the hole-drilling and camera motion.
*IC*2	Image coordinate system after the hole-drilling and camera motion.
*s*	Scaling factor that holds the third component (*w*) of the homogeneous representation {*x*, *y*, *w*}^T^ in 1.
*H_IC_*_1__→*IC*2_	Homography transform from *IC*1 to *IC*2 associated with 6DOF camera motion.
*H_h_*	Principal homography transform.
*H*	*H*_*IC*1__→*IC*2_*H_h_*.
